# Systematically developing a family-based health promotion intervention for women with prior gestational diabetes based on evidence, theory and co-production: the Face-it study

**DOI:** 10.1186/s12889-021-11655-2

**Published:** 2021-09-03

**Authors:** Helle Terkildsen Maindal, Anne Timm, Inger Katrine Dahl-Petersen, Emma Davidsen, Line Hillersdal, Nanna Husted Jensen, Maja Thøgersen, Dorte Møller Jensen, Per Ovesen, Peter Damm, Ulla Kampmann, Christina Anne Vinter, Elisabeth Reinhardt Mathiesen, Karoline Kragelund Nielsen

**Affiliations:** 1 Department of Public Health, Aarhus University, Aarhus, Denmark; 2 Health Promotion Research, Steno Diabetes Center Copenhagen, Gentofte, Denmark; 3 Department of Anthropology, University of Copenhagen, Copenhagen, Denmark; 4 Steno Diabetes Center Odense, Odense University Hospital, Odense, Denmark; 5 Department of Gynaecology and Obstetrics, Odense University Hospital, Odense, Denmark; 6 Department of Clinical Research, University of Southern Denmark, Odense, Denmark; 7 Department of Obstetrics, Aarhus University Hospital, Aarhus, Denmark; 8 Center for Pregnant Women with Diabetes, Department of Obstetrics, Rigshospitalet, Copenhagen, Denmark; 9 Department of Clinical Medicine, University of Copenhagen, Copenhagen, Denmark; 10 Steno Diabetes Center Aarhus, Aarhus, Denmark; 11 Center for Pregnant Women with Diabetes, Department of Endocrinology, Rigshospitalet, Copenhagen, Denmark

**Keywords:** Complex intervention, Health promotion, Co-production, Family intervention, Gestational diabetes mellitus, Type 2 diabetes prevention, Postpartum period, Intervention development

## Abstract

**Background:**

Women with prior gestational diabetes mellitus (GDM) are at high risk of developing type 2 diabetes; however, this risk can be reduced by engaging in positive health behaviours e.g. healthy diet and regular physical activity. As such behaviours are difficult to obtain and maintain there is a need to develop sustainable behavioural interventions following GDM. We aimed to report the process of systematically developing a health promotion intervention to increase quality of life and reduce diabetes risk among women with prior GDM and their families. We distil general lessons about developing complex interventions through co-production and discuss our extensions to intervention development frameworks.

**Methods:**

The development process draws on the Medical Research Council UK Development of complex interventions in primary care framework and an adaptation of a three-stage framework proposed by Hawkins et al. From May 2017 to May 2019, we iteratively developed the Face-it intervention in four stages: 1) Evidence review, qualitative research and stakeholder consultations; 2) Co-production of the intervention content; 3) Prototyping, feasibility- and pilot-testing and 4) Core outcome development. In all stages, we involved stakeholders from three study sites.

**Results:**

During stage 1, we identified the target areas for health promotion in families where the mother had prior GDM, including applying a broad understanding of health and a multilevel and multi-determinant approach. We pinpointed municipal health visitors as deliverers and the potential of using digital technology. In stage 2, we tested intervention content and delivery methods. A health pedagogic dialogue tool and a digital health app were co-adapted as the main intervention components. In stage 3, the intervention content and delivery were further adapted in the local context of the three study sites. Suggestions for intervention manuals were refined to optimise flexibility, delivery, sequencing of activities and from this, specific training manuals were developed. Finally, at stage 4, all stakeholders were involved in developing realistic and relevant evaluation outcomes.

**Conclusions:**

This comprehensive description of the development of the Face-it intervention provides an example of how to co-produce and prototype a complex intervention balancing evidence and local conditions. The thorough, four-stage development is expected to create ownership and feasibility among intervention participants, deliverers and local stakeholders.

**Trial registration:**

ClinicalTrials.gov NCT03997773, registered retrospectively on 25 June 2019.

## Introduction

Gestational diabetes mellitus (GDM) predisposes women and their offspring to a range of short- and long-term morbidities, including early onset type 2 diabetes mellitus (T2DM) and cardiovascular disease [1–5]. In Denmark, the prevalence of GDM is increasing and is now around 5% [6], making it one of the most common medical conditions during pregnancy. Women with prior GDM have a nearly 10-fold increased risk of developing T2DM [7] and their offspring have an almost 8-fold increased risk of developing T2DM or prediabetes in later life [4]. Further, it has been shown that partners to women with GDM have a 33% higher diabetes incidence compared to partners where the woman was not diagnosed with GDM during pregnancy [8], suggesting that both shared environment and health behaviours may contribute to making the partners susceptible to diabetes [8].

Evidence from the US Diabetes Prevention Program (DPP) suggests that intensive lifestyle intervention can reduce the risk of T2DM among women with prior GDM [9]. However, study participants in the DPP GDM sub-group analysis were, on average, 12 years beyond their first GDM affected pregnancy [9]. As the cumulative incidence of T2DM in women with prior GDM increases substantially within the first 5 years after delivery [10], there is a need to identify effective interventions in this time-period.

Previous research shows that in everyday real-world settings, changes in health behaviour are difficult to obtain and sustain. Observational studies have shown that many women with prior GDM do not follow recommendations for healthy diet and physical activity following delivery [11, 12]. Existing evidence also suggests that women with prior GDM face multiple barriers to sustaining healthy behaviours after delivery, including barriers related to everyday life with an infant or small child and lack of social support [13, 14]. These barriers tend to be interlinked and interact on several levels, e.g. individual, family, community levels [14]. Thus, it is vital that health promotion efforts are based on a thorough understanding of the barriers to healthy behaviours and involve carefully tailored solutions to overcome these barriers. Any intervention needs to be complex and context-specific in order to stand a chance of success. Evidence on how to develop sustainable behavioural interventions and how to ensure high uptake among this target group is scarce. Few studies report on the process of developing such interventions. This is likely to impact on the potential success of interventions, both in terms of implementation and sustainability.

The UK Medical Research Council (MRC)‘s framework is a widely adopted framework within complex intervention development and evaluation research in primary care, which outlines key elements of the process from intervention development to implementation [15]. However, this framework provides limited guidance on the details of the intervention development phase [16]. In our search for a comprehensive framework to support the process of intervention development among women with prior GDM, we identified the framework presented by Hawkins and colleagues for developing complex interventions [17]. The key features of the Hawkins framework include the use of comprehensive evidence review, co-production and prototyping. The three-stage framework provides examples from two interventions of how intervention content and delivery methods can be adapted and developed in an iterative and cumulative process involving external partners [17]. Involvement of a combination of the target group, key stakeholders and intervention deliverers, e.g. through co-production, has been shown to improve adaptation and the tailoring of intervention content to a specific context [18, 19], and for ensuring ownership [20]. Taken together, we draw on elements taken from these two frameworks by MRC and Hawkins et al. to systematically develop a sustainable intervention targeting women with prior GDM and their families.

In this paper, we report on the process of systematically developing a health promotion intervention (The Face-it intervention) to increase quality of life and reduce diabetes risk among women with prior GDM and their families. We present key lessons at each stage of developing complex interventions and compare our approach to other intervention development frameworks.

## Methods/design

**Fig. 1:**
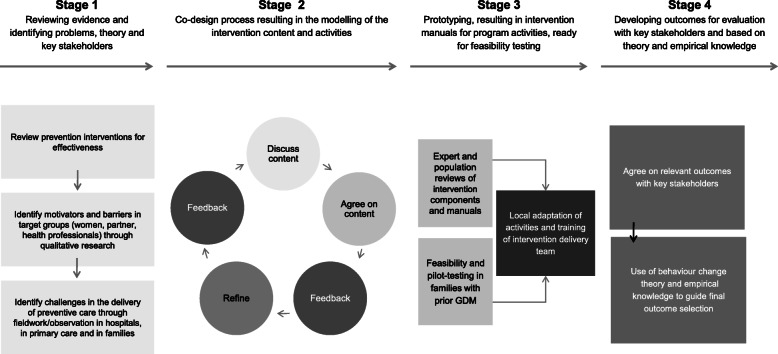
Four-stage process of the Face-it intervention development

Based on elements from the frameworks by MRC and Hawkins et al. and with the involvement of key stakeholders throughout, we developed the Face-it intervention in a fourstage process in the period from May 2017 to May 2019 (Fig. 1): (1) evidence review, qualitative research and stakeholder consultations, (2) co-production processes with families where the mother had prior GDM and health professionals (3) prototyping, feasibility and pilot testing; and, (4) developing core outcomes for the evaluation. Table 1 gives an overview of the stages and the data sources used.

**Table 1:** Key lessons from stage 1–4

Stages	Intervention content	Intervention delivery	Data sources
**Stage 1: Evidence review, qualitative research and stakeholder consultations**	No intervention type identified as superior	Initiate intervention approximately 3 months after delivery	Systematic review: Behavioural interventions targeting women with prior GDM [21]
	Targeting multiple barriers and determinants for health behaviour social support, motivation, self-efficacy, risk perception and health literacy	Multilevel strategy targeting the individual, family and health system level	Systematic review: Barriers and determinants for GDM health services and postpartum follow-up [13] Scientific symposium with experts [22]
	Not assigning blame or medicalising women with prior GDM		Qualitative study: Danish women with prior GDM to understand the needs and barriers of women with prior GDM (*n* = 6) [23]
	Partner involvement to ensure social support / improve intervention uptake and address own risk	Include the whole family as the target group	Qualitative study: Partners of women with prior GDM (*n* = 5) [24] Scientific symposium with experts [22]
	Secure a coherent healthcare system to align knowledge transfer and collaboration across sectors and create a coherent preventive pathway		Qualitative study: Healthcare professionals caring for women with GDM during and after pregnancy (*n* = 9) [25]
	Relationship with health visitor* imperative to talk about healthy habits in the family Intervention needs to be tailored and adapted to individual needs in the family, daily family life (role modelling) and based on a broad positive health concept Need for education to health visitors addressing risk behaviours and prevention	Health visitors as main intervention deliverers	Workshop and interviews with families where the mother had GDM (*n* = 5) Focus group discussions with teams of health visitors (*n* = 8) Scientific symposium with experts [22] Expert consultations
	Women expect a digital component to increase engagement and availability Introducing the LIVA app as an intervention component Digital support as a way to prompt individual and family-based health behaviours	Digital health coaching	Evidence from literature on the potential of digital interventions targeting women with prior GDM Scientific symposium with experts [22]
**Stage 2: Co-production of the intervention**	Coherent cross-sectional preventive care pathway for the families Women recommended to contact their own GP for GDM counselling following the intervention	Discharge summary from obstetric department to health visitors prior to intervention	Workshop meetings with local stakeholders and hospital-based health-care professionals from the obstetric departments at the project hospitals, general practices and leading health visitors (*n* = 3)
	Adopt the family wheel as an interactive health pedagogic dialogue tool Health visitors take on a health promoting role	Home visits to the families by the health visitor as a primary component of the intervention	Meetings with leading health visitors (*n* = 3)
	Adapting the family wheel to support talking about future diabetes risk Adapting the digital health app, making health information available in the app to use for counselling and produce family tailored content in the app	Digital coaching by a digital supervisor should motivate realistic, positive goals in the family	Co-production workshops with health visitors (*n* = 2) Interviews with families where the mother had prior GDM (*n* = 3)
**Stage 3: Prototyping, feasibility and pilot testing**	Health coaches tailoring health information per request from families Possibility for continuously digital communication with the family online instead of home visits Ensure strong communication practices between health coach and health visitor	Adaption of intervention delivery mode to intervention sites	Meetings with local health visitors and GDM experts (*n* = 4) Interviews with families where the mother had prior GDM (*n* = 4)
	Adapt intervention manuals to support individual practices Ensure proper training and competences for intervention deliverers Adaption of family wheel design	Support and qualify health visitors to deliver the intervention	Expert review of the intervention manuals by researchers, health visitors as intervention deliverers and various health care professionals providing care to women with current and prior GDM Training days with health visitors
**Stage 4: Involvement in developing outcomes for evaluation**	Realistic and relevant core outcomes for evaluation Biochemical measurements (blood samples), blood pressure, anthropometric measures and a self-administrated questionnaire to assess dimensions of health behaviour, social support, motivation, program delivery and family dynamics among others The questionnaire contained both validated scales and self-constructed questions building on the qualitative evidence from the earlier stages of intervention development The full list of measurements is available in the trial protocol [26]		Core outcome set for diabetes after pregnancy prevention [27] Based on the core outcome set, the qualitative interviews performed at stage two and the consensus meetings with core stakeholders (*n* = 130)
	Implementing minor adjustments to the questionnaire to avoid assigning blame or stigmatisation and to enhance validity		Pilot testing of the questionnaire among women with prior GDM (*n* = 5)

*Specialised nurses within postnatal and child health, who conduct home visits to families with a new-born

### Stage 1: evidence review, qualitative research and stakeholder consultations

The first stage was conducted to gain a thorough understanding of the existing evidence relating to health promotion and prevention in the target group, including the needs and barriers for health behaviour change and postpartum follow-up experienced by women with prior GDM. We also wanted to identify avenues for sustainable health promotion initiatives, not only for the women as individuals, but as part of a larger social system, including their partner and the health system [22]. We carried out two systematic literature reviews. The first review investigated RCTs of behavioural interventions aiming to prevent T2DM in women with prior GDM implemented in the first 2 years after delivery [21]. The second review focused on the determinants and barriers to accessing GDM services, including postpartum follow-up [13]. Both studies, with detailed descriptions of methodology, have been published elsewhere.

To understand the needs and barriers of women with prior GDM, their partners and healthcare professionals in the Danish setting, we conducted three small-scale explorative qualitative studies, of which two have been published [23, 25]. Specifically, we adopted an explorative approach and used self-determination theory [28] and the Behaviour Change Wheel [29] to uncover behavioural determinants such as motivation, health literacy, self-efficacy, social support and risk perception in an intervention context. We conducted semi-structured interviews with open-ended questions with six women with GDM, five partners of women with GDM, and 10 healthcare providers working with GDM. Participants for the interviews were recruited via nurses and physicians at obstetric departments, social media and through the use of snowball sampling, where research participants help recruit other participants. Interviews were audio recorded, transcribed verbatim and coded following thematic content analysis. From these studies, it became apparent that health visitors were particularly well-suited for the role as intervention deliverers. In Denmark, all women who have given birth are offered home visits by a health visitor (specialised nurses within postnatal and child health). These health visitors are based in the local municipality and have unique access to families and their everyday life. A key role of health visitors is to provide guidance on the health and well-being of the new-born and the family.

Finally, we investigated potential avenues for health promotion initiatives among those who would deliver the intervention and the target group. This included consultations and discussions with experts in the field, such as obstetricians, endocrinologists, epidemiologists as well as national and international researchers with experience in conducting related interventions. We also organised a scientific symposium at which existing and emerging evidence was presented and discussed along with ideas for future interventions [22]. Two focus group discussions with teams of four health visitors (*n* = 8) (across two of the municipality sites where the intervention was expected to be delivered) to explore the most appropriate intervention sites, staff and activities for a behavioural intervention. We also carried out a workshop with two families and interviews with three women with prior GDM focusing on their perceptions of the GDM diagnosis, risk of diabetes, health in the family and support via healthcare providers.

### Stage 2: co-production of the intervention

Drawing on the evidence and knowledge gathered at stage 1, we established an intervention development group consisting of members of the Face-it research group and two leading health visitors. Their remit was to adapt existing evidence and co-produce new intervention activities. We held a workshop with relevant healthcare professionals, including health visitors, to test ideas generated in the first stage. The workshop also explored experiences with current and prior health promotion initiatives and interdisciplinary collaborations. To ensure involvement of the target group at this stage and to verify the tentative notions about motivators for change and ideas for intervention components that emerged during stage 1, we interviewed four families and three women with prior GDM. These were recruited through diabetes nurses conducting follow-up group consultations after delivery and through health visitors hosting baby swimming to women with BMI ≥ 27 kg/m^2^ in pregnancy. These interviews addressed issues uncovered in the workshops relating to maintaining healthy living after delivery.

### Stage 3: prototyping, feasibility and pilot testing

In this stage, the intervention components identified in the previous stages were tested separately with both families and health visitors. By the end of this stage, we finalised a programme theory and an intervention manual with associated resources. We held four training days for the 12 health visitors, who were going to deliver the intervention. Further, one health coach participated as she was supposed to be responsible for the digital coaching at one of the sites. At the training sessions, the participants tested parts of the intervention for feasibility and provided ideas and inputs to adapt the intervention manuals. The participants were educated to take on the role as digital health coaches with a specific focus on dietary and physical activity behaviours. By the end of stage 3, the draft intervention manuals and associated resources underwent expert review by the Face-it research group, the health visitors and the clinicians involved in providing care for women with current and prior GDM. All of them were from the three areas where we planned to conduct a later trial.

### Stage 4: involvement in developing outcomes for evaluation

At the fourth stage, which is an add-on to the well-known MRC framework [15], we sought to develop realistic and relevant health promotion outcomes for the evaluation of the study with the involvement of researchers, clinicians and women diagnosed with GDM. Together with international collaborators, we used established methodologies to develop a core outcome set, i.e. a standardised set of outcomes to be reported across trials within a specific area [30]. The methods for this stage have been published in detail elsewhere [31]. In brief, this stage included 1) a systematic literature review to identify outcomes used in existing intervention studies, 2) an investigator meeting to discuss and clarify the review findings, 3) a two-round e-Delphi survey where women with current or prior GDM, health care/public health professionals and researchers were asked to assess and prioritise the identified outcomes, and 4) nominal group consensus meeting with key stakeholders where the e-Delphi survey results were discussed and the top-rated outcomes appraised and prioritised for inclusion in the core outcome set using a voting system.

Following the development of the core outcome set, we consulted various experts and carried out consensus meetings within the Face-it group to decide on the full set of outcomes and data variables to be collected for the evaluation of the Face-it intervention. Further, we pilot tested the questionnaire among women with prior GDM to achieve face and content validity.

## Results

Table 1 gives an overview of the key lessons derived from each of the four stages in the development of the Face-it intervention.

### Stage 1 evidence review, qualitative research and stakeholder consultations

Our systematic review of RCTs evaluating behavioural interventions that aimed to prevent T2DM in women with prior GDM [21] identified 10 trials which examined the effect of the intervention on various metabolic indicators. The included studies were limited by small sample sizes and substantial heterogeneity in both intervention components and outcome measures. This complicated firm conclusions about the superiority of specific intervention content, duration or modes of delivery. Therefore, based on the included studies, it was not possible to identify one specific intervention type as superior, but meta-analysis of four trials assessing the effect on diabetes incidence showed that interventions in the first 2 years after delivery were superior to no intervention (pooled estimated of risk difference per 100: − 5.02 (− 9.24;-0.80)). Furthermore, there was a tendency for interventions that started during pregnancy or within the first 6 weeks after delivery to have poorer outcomes compared to interventions starting later. This informed our decision to initiate the Face-it intervention approximately 3 months after delivery, which would also allow the baseline data collection for the trial evaluation to align with the timing of the routinely recommended postpartum oral glucose tolerance test.

Our systematic review that explored determinants and barriers for GDM services, including healthy lifestyle after delivery and prevention of future T2DM [13], identified risk perception, self-efficacy and social support as important determinants for engaging in healthy dietary and physical activity behaviours. Consequently, these constructs became determinants that we sought to promote through the intervention. The review also identified a number of barriers, such as lack of time, motivation and social support, and suggested that women with prior GDM may be facing emotional distress.

The review on determinants and barriers predominantly identified studies from the U.S., Canada and Australia. However, the qualitative studies we performed gave us evidence from the local Danish context in which our intervention would be carried out and evaluated. The first of these explored the experiences of five women with previous GDM within the first 3–4 months after the delivery [23]. The women in the study described emotional distress as a consequence of the GDM diagnosis, which was similar to the findings of the systematic review. Danish women with prior GDM reported feelings of sadness, guilt and self-blame, and it was apparent that the intervention needed to be sensitive to these feelings and not to assign blame to the mother or induce medicalisation.

The women in our explorative qualitative study also emphasised the importance of social and emotional support in general, and particularly from their partners, to mobilise time and energy to follow a diet and physical activity regime [23]. This coincided with a postpartum intervention study from the UK and Canada, which showed that not only did paternal weight correlate with maternal and offspring weight, but having a partner involved in the study was associated with successful study completion [32]. This convinced us that we needed to include the partner in our study, both to address his/her cardio-metabolic risk and as a source of social support for the woman with prior GDM. To further examine how this might manifest in a Danish context, we interviewed five male partners of women with prior GDM [24]. A key finding from these interviews was that the baby and the family have absolute first priority. Therefore, taking time to, e.g. exercise, was perceived as selfish and associated with feelings of guilt. However, being a good role model for one’s child by being physically active was also highlighted in the interviews. The challenge was thus to create an intervention which promoted healthy behaviours in the context of being a good role model rather than taking away precious time from the family.

Our review also indicated that a lack of knowledge about the risk of T2DM after the diagnosis of GDM and guidelines for health service support were a barrier to sustaining a healthy behaviour after delivery [13]. Furthermore, women considered postpartum health services to be unsupportive and most women were not aware of postpartum services or did not know how to navigate them:

*“I’ve been to my doctor and had my blood sugar tested, but then there are no more [follow-up] after the delivery. I just think it’s easy to fall back into the unhealthy lifestyle again when there isn’t anyone keeping an eye on you anymore [ … ] You are a bit abandoned and left on your own when you’ve delivered” (Woman with prior GDM, quote from Svensson et al* [23]*)*

This finding suggested that poor health literacy and challenges in accessing the healthcare system required further exploration. Therefore, we conducted a third qualitative study; this time focusing on healthcare providers and the health system level [25]. The study showed that health visitors, despite playing a key role in health promotion in families in the first years of the baby’s life, had limited knowledge about GDM and its implications. Often, the health visitors were not even aware whether a woman had been diagnosed with GDM or not. Findings also suggested that general practitioners (GPs) often omitted follow-up and long-term risk measurement after GDM. Moreover, we discovered that women received opposing messages from different healthcare providers, which could lead to women neglecting their long-term risk of diabetes. Thus, it was apparent that increasing health visitor skills and knowledge about GDM was required and that knowledge transfer and collaboration across sectors needed to be established to align knowledge about GDM and create a coherent preventive pathway.

From the scientific symposium [22] a key recommendation was to apply a multi-determinant approach and structure the intervention on multiple levels. For example, it was agreed that barriers to healthy behaviour exist and should be addressed at the individual, family and health system levels. Further, it would be necessary to take on a broad and positive understanding of health in line with the WHO definition focusing on social, psychological and physical health [33].

Our consultations and workshops with midwives and health visitors further strengthened health visitors’ potential as the most optimal group of intervention deliverers. In particular, health visitors provide counselling based on the broad WHO concept of health. However, the consultations and workshops also confirmed the qualitative research findings that the health visitors needed additional training. They were not particularly comfortable with addressing risk behaviours and disease prevention in our target group:

*“We don't come into parents’ home with a raised finger. And if one can see that there are a lot of soft drinks on the table in a home, then we may address this in a broader way by paying attention to food and meals in general terms”* (Health visitor)

At the symposium, experiences from the Australian MAGDA study demonstrated that a tele- or digital component might hold promise as an approach to improve engagement in the intervention among women with prior GDM [34, 35].

In addition, other studies have identified app-based technology as a possible solution to support people at risk of diabetes [36] and women with prior GDM in particular [37, 38]. One argument is the flexibility that such eHealth solutions offer as they can be accessed in people’s own homes and at any time of the day. In this way, eHealth technologies can increase the availability of health promotion to populations that are usually difficult to reach [39, 40]. We decided to further explore the potential for involving a digital solution and found a few digital platforms incorporating the interpersonal level, e.g. relying on social support and feedback, which was suggested by the women in our workshops. We identified the Liva app as the best e-solution for adaptation and tailoring to the families in the Face-it intervention [41, 42]. The Liva app is an interactive eHealth lifestyle coaching program (long-term Lifestyle change InterVention and eHealth Application [LIVA 2.0]) [43]. The app builds on a strong personal relationship between user and a health coach, who supports the user through individualised goal-setting and feedback [43]. As digital support was suggested by the women themselves in initial interviews, we found that the combination of providing digital support as an addition to home visits aligned with the tailoring of intervention to meet the needs of the target group. The health visitors involved in this stage reported that they could take on the role as health coach as well.

From stage 1, we identified health visitors as intervention deliverers in the family and found a digital, interactive platform as part of the intervention content. As such, we left an exploratory phase and proceeded into co-production to identify practical solutions for intervention content.

### Stage 2 co-production of the intervention

The second stage in the systematic development process was based on continuous development and adaptation of the knowledge gained in stage 1, and aimed to design an intervention prototype that was ready for testing [15]. Together with health visitors, families and hospital-based healthcare professionals involved in GDM care, we co-produced the intervention content and delivery components: Specifically, we presented the findings from stage 1 through workshops as well as the intervention premises to co-develop potential intervention content to accommodate the families’ barriers and motivators for healthy behaviour. Further, we had health visitors suggesting their own available resources for adaptation and initiated role play exercises to tailor identified intervention tools. In particular, we wanted to ensure a smooth and coherent transition from hospital discharge after delivery to the health promotion intervention delivered by municipal health visitors.

#### The cross-sectoral preventive pathway

To ensure a coherent cross-sectoral preventive pathway for the women, both in the trial and in a possible future implementation, the local stakeholders from the three project hospitals, general practices and senior health visitors were invited to local workshops to discuss possible care pathways. The healthcare professionals in the hospitals were satisfied with systematic information flow across professions in the obstetric department. However, GPs and health visitors felt limited by the lack of information provided to them by the obstetric departments.

We interviewed women and their families about their experiences of GDM-related care among other topics. The women described a need to leave the GDM diagnosis behind due to the strict treatment regimen they experienced in pregnancy. However, the families also recognised the benefit of the health visitor taking on a health promotion role to motivate health behaviours in the family.

*“It is very important that it does not become a raised index finger but becomes motivating. So, you think to yourself "that was a good idea". I think it depends a lot on how your relationship with the health visitor is”* (Partner to woman with prior GDM)

We returned to the healthcare professionals with new insights from the families and considered the best ways to secure knowledge transfer from obstetric departments to municipal health visitors. They suggested providing a hospital discharge summary to the health visitor delivering the intervention. In order to create a coherent pathway for the families, the health visitors also suggested that they, by the end of the intervention, should encourage the women to book and attend the recommended glucose test and counselling with her GP. The idea was that this would strengthen communication and knowledge transfer to the GP and would increase the likelihood of the women being followed-up regularly by their GP as recommended. Thus, engaging closely with the health visitors and hospital staff allowed the identification of a possible solution for a coherent care pathway that lived up to the requests of all stakeholders.

#### Home visits and an interactive dialogue tool: ‘the family wheel’

We met with the health visitor management in one of the three municipalities that we planned to involve in a later trial and presented our current principles on how to promote health in families where the mother had GDM, e.g. focusing on the broad health concept of WHO, social support, motivation, self-efficacy, risk perception and health literacy [26]. This led the health visitors to introduce a health pedagogic tool: *the family wheel*. The *family wheel* is an interactive dialogue tool developed by health visitors themselves to support socially vulnerable families in the transition to parenthood both during and after pregnancy. A prior evaluation of the family wheel found that health visitors used it to help structure and professionalise their dialogue with families. The family wheel originally contained relevant themes for a postpartum intervention, including social relations, breastfeeding, living situation etc. We saw great potential in modifying this conversation tool to uphold the health visitors’ usual practice and structure their new role as health promoters for the whole family. Earlier interviews with families had taught us that a close relationship between the families and the health visitor was critical to enable an open conversation about health, particularly as this often involved sensitive topics, such as overweight, future diabetes risk, partner support and specific food and physical activity habits. In workshops with health visitors, we discussed how the increased risk after delivery could be presented in a motivating way by using the family wheel. The health visitors were not used to addressing parents’ health behaviour and expressed concerns about unintendedly stigmatising the families. Yet, health visitors suggested that by adopting the family wheel as part of standard practice in the intervention, it legitimised conversations on health risk which led to the first thematic category on the modified family wheel: ‘GDM’. The main topic would be a debriefing of the experiences from the GDM-affected pregnancy and a discussion on the risk of T2DM. When asking the health visitors how to modify the wheel further, they specified the need to touch upon all themes relevant to health:

*“When I set it [the family wheel] up, I usually ask them how much they need to talk about that theme. The area in question is pointed out. And I do not follow the manual slavishly. Because it may well be that they have no need to talk about gestational diabetes but have a huge need to talk about childbirth. It may be easier to articulate some topics and to get into some issues if they suggest it themselves”* (Health visitor)

It was essential to the health visitors to make the families reflect on their health views and encourage already established health behaviours. We redesigned the wheel through continuous dialogue with the health visitors. The family wheel finally included five topics: 1) GDM, 2) everyday routines, 3) food and meals, 4) physical exercise, and 5) family, friends, and network. As such, health in the family was the main focus and GDM was only one in five themes of the family wheel to be addressed. When we presented the family wheel to the families, they were satisfied with the broad aspect of topics and did not feel that they were defined only by their GDM diagnosis. By making health comprise of multiple and interconnected areas, the families perceived this part of the intervention as welcoming a focus on their daily lives.

The choice to adopt the family wheel in the Face-it intervention helped facilitate a strong collaboration with health visitors. Health visitors expressed ownership across municipalities as they felt acknowledged in their profession by building on similar pedagogical non-directive and non-judgmental methods and gained new knowledge about this high-risk group. Moreover, it strengthened the methodological quality of the intervention by tailoring and qualifying the material to their profession. In this way, the adoption of new themes into the family wheel supported health visitors in taking on a new role as health promotors. They helped the families to navigate health information and services, thus increasing health literacy and facilitating and increasing positive family dynamics and social support around health behaviour change.

#### Digital health promotion counselling through ‘the Liva app’

As a result of the findings from stage one, we wanted to introduce the Liva app as part of the intervention content to families and health visitors. The Liva app includes health behaviour features; however, it was clear that the content was shaped by other target groups e.g. those with diabetes or overweight who would report on medication use and blood sugar values [43]. When introducing the app to the health visitors, they were less enthusiastic about the digital solution. The health visitors would usually spend time during home visits encouraging families to reduce their screen time and they felt ambivalent towards promoting an app. As such, the health visitors emphasised the need for the app to promote positive everyday activities:

*“It [the app] should follow up on what succeeded for you and not what failed. Because I may have a goal to “run on Wednesday afternoon”, but it did not work out … And I do not think they would benefit from that at all. But look, I went Monday!” (Health visitor)*

Through co-production with health visitors, we emphasised the role of the health coach to ensure that the families would set goals based on the families’ own wishes, preferences and circumstances. Thus, we decided that the built-in feature of ‘life goals’ should be highlighted in the digital support as a way to prompt individual and family-based health behaviours. A goal could be to ask a friend to go for a walk, read a book, create a shopping list, plan the snacks for the day, or to encourage your partner to go for a walk etc. In accordance with the families’ wishes for an app, a breastfeeding feature was developed, and to accommodate a broader understanding of exercise, the category of physical activity was expanded to include activities drawn from everyday life in a family i.e. activities such as ‘walking’, ‘vacuuming’, ‘exercises with baby’ or ‘gardening’.

The Liva app also helped counter some other challenges raised by health visitors at this stage. The health visitors were worried about their ability to provide specific advice on GDM, diet or exercise if requested by the families. By making specific health information available in the app, we wanted to assure the health visitors that they were not expected to be experts in all health-related topics. We tailored materials in the app to families of women with former GDM, such as physical activity and dietary recommendations, exercise charts and shopping lists etc. To finalise the content, we wrote manuals for the family wheel and the Liva app and started recruiting health visitors in the three municipalities.

The co-production phase was finalised as the intervention was now premised by health-visitor-led home visits guided by the family wheel and a tailored health app, and an intersectoral knowledge pathway. We ended the co-production phase when the Liva app and family wheel were approved by health visitors.

### Stage 3 prototyping, feasibility and pilot testing

In stage 3, the core intervention components in the Face-it intervention were ready for modelling and testing as a whole in the municipalities. At this stage, we involved families, GDM experts and health visitors and health coaches (in two of three settings, this was the health visitor) as intervention deliverers aiming to 1) secure testing and tailoring of content, 2) strengthen ownership, 3) adapt intervention delivery to the local context and 4) ensure proper training and competences.

We tested the acceptability of the family wheel and Liva app with two families and two women with prior GDM. In these interviews, we addressed topics on the family wheel e.g., ‘food and meals’ and ‘exercise’ to test the acceptability by enquiring into how families experienced talking about these subjects and asked whether they felt comfortable with talking to a health visitor about this. The family wheel was assessed to be acceptable while relying on only a few contextual factors. Firstly, its aim to address sensitive subjects in the family depended on a trusting relationship between the family and the health visitor. Secondly, the fact that health visitors would come to the participants’ homes provided more flexibility for the families as they did not have to transport themselves. Thirdly, the families noted a concern regarding the Liva app about the time needed for data registration and the app potentially competing with other digital elements, e.g. watches with step counts. This concern about the app was balanced by the families’ positive attitude towards their ability to easily access a health visitor/health coach and the possibility of receiving tailored health information, e.g. in the form of recipes.

*“I would think it would be a good idea that someone is pushing me to do it. But I don’t think my husband would use it at all. I think I would choose something like exercise, weight, or diet in the app. My milk production is not very good so it could be very nice to talk about what could help increase it [through the app]”* (Woman with prior GDM)

We then held meetings with each municipality to tailor the structure of the intervention to local resources and preferences. The local municipalities decided themselves how to organise the staff delivering the intervention.

GDM experts (dieticians, nurses, endocrinologists and obstetricians) from the collaborating hospitals were invited to discuss the intervention components and adapt the cross-sectoral pathway with senior health visitors to ensure a coherent preventive pathway at the three intervention sites. The experts raised the issue that women with prior GDM and their partner often varied in their perception of GDM. In contrast, others emphasised the role of inactivity and poor diets and dealt with lack of motivation to change health behaviours. Further, the duration and frequency of the intervention with three home visits within 9 months was deemed appropriate by health visitors and experts as long as the health coaching was available between the visits to provide feedback and advice. Thus, the delivery of the intervention demanded continuous tailoring of communication to meet the needs of the families and ensure the intervention deliverers collaborated with the families to support the achievement of behavioural goals.

Lastly, we conducted four full training days for health visitors/health coaches to educate them in intervention delivery. At these training days, we presented the intervention manual consisting of a conversation guide for each theme on the family wheel. Thereafter, the education was problem-based, e.g. the health visitors pointed towards three challenges after the first day’s training with the family wheel: balancing the conversation of future risk in the family; engaging the partner in the home visit, and getting the families to act on their goals. These three themes became central to the following two training days. One training day was exclusively focused on using the Liva app. Throughout the training days, we pilot-tested the home visits in the intervention by using case descriptions of various families, probing communication strategies and adding suggestions for ‘good questions’ to start a conversation in the intervention manual. The health visitors found that the visual design of the family wheel, including the use of colours (green, yellow, red), helped them approach certain topics, but also helped the families to assess their own wishes for change within those topics. Some flexibility was allowed in terms of which theme to talk about when and in the approach to addressing topics and posing questions.

### Stage 4 involvement in developing outcomes for evaluation

In the development of a core outcome set for health promotion in diabetes after pregnancy, the 115 key stakeholders agreed on 19 relevant themes during the final consensus meeting. Core outcomes for the specific intervention depended on the focus of the intervention. These included constructs from behavioural change theory (self-efficacy, motivation, barriers and perceived risk), health behaviour (dietary intake, physical activity, sleep and breastfeeding), cardio-metabolic- and adiposity measures (body mass index, weight, waist circumference, glucose, cholesterol, and blood pressure), offspring outcomes (growth, diabetes), quality of life, knowledge, social support, and program delivery (participation, engagement). The detailed description of the core outcome set has been published elsewhere [27, 31, 44]. The involvement of different stakeholders in selecting the outcomes allowed for the inclusion of different perspectives on what was considered important to measure. Particularly, including women with GDM in the process meant that more ‘patient-oriented’ outcomes, e.g. social support and quality of life, were retained in the core outcome set [27].

Based on the core outcome set, the qualitative interviews performed at stage two and the consensus meetings with core stakeholders, the research team made the final decisions on which outcomes to include in the evaluation of the Face-it trial. Data collection covered: biochemical measurements (blood samples), blood pressure, anthropometric measures and a self-administrated questionnaire to assess dimensions of health behaviour, social support, motivation and family dynamics. The questionnaire contained both validated scales and self-constructed questions building on qualitative evidence from the earlier stages of intervention development. The full list of measurements is available in the trial protocol [26]. The findings from the previous stages informed the need to include various psychosocial outcomes. The finding that emotional distress was often present in the target group after delivery combined with the results from the core outcome set informed the decision to include questions on quality of life. The findings about the importance of motivation, risk perception, family dynamics and partner support for health behaviour in the child’s first year formed the decision to construct a set of questions on health behaviours in the family context. We also needed to investigate stigmatisation in relation to GDM diagnosis. Therefore, we developed and pilot-tested a new scale to investigate internalised stigmatisation related to GDM. After identifying the outcomes and finalising the intervention content and modes of delivery, we estimated sample size as well as recruitment and retention rates and finalised the study protocol. The details are available in the published study protocol [26].

## Discussion

### Brief summary of overall findings

This study contributes to the evidence base of how to optimise the prevention of T2DM after GDM and the challenge of creating a health promoting and preventive care pathway across established health sectors. With the use of evidence, theory and co-production, we identified and tailored the Face-it intervention for families after a GDM-affected pregnancy. Learnings from each stage framed the overall intervention approach, which is built on a broad, positive health concept, multi-level components and embedded in multi-level supportive environment. The process was immensely complex and both time- and resource consuming. We allowed the iterative processes to last for 17 month before moving into the trial phase. The process involved stakeholders across three parts of the Danish healthcare system. The cross-sectional approach involved the reconciliation of strong and sometimes opposing views and it challenged healthcare professionals’ views of their role. Nevertheless, this process seems very promising for future collaborating across sectors, and it is likely to increase uptake and positively impact outcomes for the local delivery of this complex intervention.

Previous behavioural intervention studies aiming to prevent T2DM among women with prior GDM have had varying success [21]. In the development of the Face-it intervention, we sought to learn from and build upon the experiences of these prior interventions. A number of features distinguish our intervention development process from earlier ones, including extensive co-production of the intervention with the target group and other stakeholders; the multilevel approach focusing not only on individual women with GDM, but also their partners, family and the health system; a broad and positive health concept addressing physical health, mental health and social wellbeing; and finally, the reliance on behaviour change theory to address determinants such as risk perception and health literacy. Taken together, this approach is expected to strengthen ownership, relevance, feasibility and engagement of the intervention among intervention deliverers and women with prior GDM and their families [32, 45, 46].

The result of our theory-based, co-produced intervention development process is a complex intervention that contains multiple components, involves multiple stakeholders, and works across several sectors of the healthcare service. While the Face-it intervention contains some ‘generic’ elements, our experience supports findings from other complex interventions that interventions need to be tailored to local and individual contexts [47]. Even in a small and homogeneous country like Denmark with a universal public healthcare system, we had to introduce site-specific adaption and tailoring across our three delivery sites. The need for such adaption may be particularly pertinent when addressing a complex health problem like GDM and T2DM prevention, where there are physical and psychosocial aspects to consider, and where cross-disciplinary and cross-sectoral collaboration is needed.

### Use of frameworks and co-production

The development of the Face-it intervention was guided primarily by combining the frameworks from MRC and Hawkins et al. [15, 17]. Together, these approaches acknowledge both the challenges of health behaviour change and that intervention planners have imperfect information from existing literature at almost all stages in the development process. The frameworks suggest iterative processes where evidence is adapted to the local context and specific target groups and allow the planners to be open-minded and flexible, while developing the optimal intervention. Although the frameworks were helpful throughout the development process of the Face-it intervention, following them was also resource- and time-consuming. A significant implication for similar future studies may be to monitor the resources used closely and include e.g. exact time spans and cost in evaluations strategies. At times, it was hard to judge when and how the processes should proceed. It was also tempting to return to former stages e.g. to conduct additional literature reviews or interviews focusing on the family level impact. Such inclinations had to be balanced with the available resources and timeline as well as other project priorities. We also observed conflicts in the intervention development process that arose from the different perspectives from different stakeholders and target groups. Untangling such conflicts and facilitating consensus and support across the range of stakeholders required additional time and patience.

At times, the iterative and co-production approach to developing the Face-it intervention was very challenging [48]. However, reflecting on our experiences and in line with Moore et al. [49], we also found that drawing on established staff and resources had the potential to create ownership. In addition, the iterative methods and co-creation approach were important for the successful development and implementation of the intervention. They were also useful for promoting cross-sectional and cross-disciplinary collaboration and to encourage uptake of the intervention. High levels of involvement have been found to benefit collaboration, impose culture change and ownership among intervention deliverers [18, 50, 51]. This is even more important to help secure acceptability and feasibility among the target group [52, 53]. Based on our experience of developing the Face-it intervention, we believe that is it imperative to include all stakeholder groups in the development of an intervention, as they each make important contributions to the intervention development process.

Since we started our comprehensive intervention development phase back in 2018, several new complex intervention frameworks or extensions to existing frameworks have been published. O’Cathain et al. [54] provide a helpful overview of the literature and divide the approaches into nine categories of intervention development: 1) partnership; 2) target population-centred; 3) theory-based and evidence-based; 4) implementation-based; 5) efficiency-based; 6) step-based or phased-based; and 7) intervention-specific; 8) combination and 9) pragmatic. The MRC framework, which we used, is an example of the ‘theory- and evidence-based‘ category. When combining the framework with the Hawkins et al framework, our work also covers the category of ‘partnership’, which is categorised by co-production. Not all of the approaches have specific guidance that describe their use and the implications of using each category remain to be elucidated. When conducting empirical work for our intervention, we faced difficulties in strictly adhering to one framework and found it useful to combine aspects from two frameworks. There is also much to be gained from publishing rich descriptions of how any published intervention development was operationalised for the purpose of creating generic knowledge and passing on valuable learning to other intervention developers.

### Future activities in the face-it study

Hawkins and colleagues note that you may have to continue to adapt the intervention even after agreeing on the content [17]. This is certainly something we have observed in the Face-it study, as we transition from the development and feasibility phases to the evaluation phase [15]. We have planned additional training days for intervention deliverers and those recruiting and conducting clinical examinations. The training is based on co-production principles, including the possibility of further adaptation of the intervention content without compromising the core of the intervention [26]. Wherever possible, the training days will ensure ongoing involvement and transparency in decisions made in the study. We will report on the challenges and learning from implementing the Face-it intervention in future papers.

## Conclusion

This comprehensive description of the development of the Face-it intervention provides an example of how to co-produce and prototype a complex health promotion intervention, balancing evidence and local conditions. In line with complex intervention theory, the Face-it intervention contains both flexible and stable intervention components and processes.

The thorough development of the Face-it intervention in four stages is expected to create ownership, relevance, feasibility and engagement among intervention participants, deliverers and local stakeholders. This is likely to increase uptake and positively impact outcomes for this complex intervention. The addition of the fourth stage on the development of outcomes with the involvement of stakeholders is expected to support an accurate and relevant evaluation of the Face-it trial. Delineation of co-produced outcomes will also contribute to a systematic approach to the heterogeneous field of studies in the field of preventing diabetes after pregnancy. The ongoing evaluations of the Face-it intervention will capture the effectiveness and the context-specific processes of the intervention. This study contributes to the evidence base of how to optimise the prevention of T2DM after GDM and the challenge of creating a health promoting and preventive care pathway across established health sectors.

## Data Availability

The datasets used and/or analysed during the current study available from the corresponding author on reasonable request.

## Abbreviations

GDM: Gestational diabetes Mellitus
GP: General practitioner
T2DM: Type 2 diabetes mellitus
